# Comparative Structural and Functional Analysis of Orthomyxovirus Polymerase Cap-Snatching Domains

**DOI:** 10.1371/journal.pone.0084973

**Published:** 2014-01-15

**Authors:** Delphine Guilligay, Jan Kadlec, Thibaut Crépin, Thomas Lunardi, Denis Bouvier, Georg Kochs, Rob W. H. Ruigrok, Stephen Cusack

**Affiliations:** 1 University Grenoble Alpes, Unit of Virus Host-Cell Interactions, Grenoble, France; 2 Centre National de la Recherche Scientifique, Unit of Virus Host-Cell Interactions, Grenoble, France; 3 European Molecular Biology Laboratory, Grenoble Outstation and Unit of Virus Host-Cell Interactions, Grenoble, France; 4 Institute for Virology, University Medical Center Freiburg, Freiburg, Germany; University of Edinburgh, United Kingdom

## Abstract

Orthomyxovirus Influenza A virus (IAV) heterotrimeric polymerase performs transcription of viral mRNAs by cap-snatching, which involves generation of capped primers by host pre-mRNA binding via the PB2 subunit cap-binding site and cleavage 10–13 nucleotides from the 5′ cap by the PA subunit endonuclease. Thogotoviruses, tick-borne orthomyxoviruses that includes Thogoto (THOV), Dhori (DHOV) and Jos (JOSV) viruses, are thought to perform cap-snatching by cleaving directly after the cap and thus have no heterogeneous, host-derived sequences at the 5′ extremity of their mRNAs. Based on recent work identifying the cap-binding and endonuclease domains in IAV polymerase, we determined the crystal structures of two THOV PB2 domains, the putative cap-binding and the so-called ‘627-domain’, and the structures of the putative endonuclease domains (PA-Nter) of THOV and DHOV. Despite low sequence similarity, corresponding domains have the same fold confirming the overall architectural similarity of orthomyxovirus polymerases. However the putative Thogotovirus cap-snatching domains in PA and PB2 have non-conservative substitutions of key active site residues. Biochemical analysis confirms that, unlike the IAV domains, the THOV and DHOV PA-Nter domains do not bind divalent cations and have no endonuclease activity and the THOV central PB2 domain does not bind cap analogues. On the other hand, sequence analysis suggests that other, non-influenza, orthomyxoviruses, such as salmon anemia virus (isavirus) and Quaranfil virus likely conserve active cap-snatching domains correlating with the reported occurrence of heterogeneous, host-derived sequences at the 5′ end of the mRNAs of these viruses. These results highlight the unusual nature of transcription initiation by Thogotoviruses.

## Introduction

Orthomyxoviruses are a family of negative strand RNA viruses with 6–8 genomic segments. The best known genus is Influenza A virus (IAV), which infects mainly water and domestic fowl although some strains cause disease in mammals such as pigs, horses, seals and humans. Other genera of orthomyxoviruses include Influenza B virus, Influenza C virus, Isavirus (infectious salmon anemia virus), Thogotovirus and Quarjavirus [1], [2]. Four species of Thogotovirus, which have six genome segments compared to eight in Influenza viruses, have been described. Thogoto virus (THOV) itself was isolated in 1960 from *Rhipicephalus sp.* ticks collected from cattle in the Thogoto forest in Kenya [3] and was later found to be widespread in Africa and southern Europe. Dhori virus (DHOV) was first isolated in India from camel ticks, *Hyalomma dromedarri* [4] but has also been detected in eastern Russia, Egypt, and southern Portugal. Both THOV and DHOV can infect humans through tick bites or accidental laboratory infections, causing febrile illness, infection of the liver and encephalitis [5]–[7]. Two other Thogotoviruses are Araguari virus (ARAV) isolated from an opossum in Brazil [8] and Jos virus (JOSV) isolated from *Rhipicephalus sp.* ticks in central Africa [9]. The distinct genus Quarjavirus includes Quaranfil (originally from Egypt), Lake Chad (originally from Nigeria) and Johnston Atoll (originally from the central Pacific) viruses [2].

Efficient translation of viral mRNAs in eukaryotic cells requires that they possess either a 5′ cap structure or special RNA structures called internal ribosome entry sites, which bypass the need for the cap. Many viruses that use capped mRNAs code for the required capping enzymes (reviewed in [10]. However this is not the case for IAV, which ‘snatches’ the cap structure from cellular pre-mRNA [11]. Bunya- and arena viruses, which also have a segmented negative strand RNA genome, follow the same strategy [12], [13]. The IAV polymerase consists of three subunits coded by the three longest gene segments; PB1 that contains the motifs for the RNA-dependent RNA polymerase activity [14], PB2 that contains the cap binding activity [15] and PA that has an N-terminal endonuclease domain [16], [17] (reviewed in [18]. PB2 also contains the so-called 627-domain, named because it contains the host specificity residue 627, which is a lysine in human IAVs and a glutamate in avian strains [19]. Cap-snatching involves firstly an independently folded domain of PB2 (residues 320–483) binding the cap structure of the cellular pre-mRNA [15]. Subsequently, a divalent cation dependent endonuclease at the N-terminal domain of PA (residues 1–197) then cleaves the mRNA at 10–13 nucleotides from the cap [16], [17]. Finally, the polymerase domain of PB1 uses this capped fragment as a primer for the transcription of viral mRNA. The resulting chimeric viral mRNAs are variable in sequence at their 5′ ends. This cap-snatching activity is thought to be regulated by the binding of the conserved 5′ and 3′ ends of the viral genomic RNA to the polymerase [20], [21]. Since cap-snatching is essential for IAV replication, the cap-binding and endonuclease domains in PB2 and PA, whose crystal structures are now known, are good targets for structure-based anti-viral drug design [22], [23].

The three largest gene segments of the Thogotoviruses, THOV, DHOV and JOSV are homologous to those of IAV and code for the heterotrimeric polymerase [9], [24]. Similar to IAV, THOV polymerase is thought to perform cap-snatching that is regulated by the terminal ends of the genomic RNA [25]. However, a major difference is that THOV mRNAs are homogeneous in length and sequence, with only the 5′ m^7^GpppA_m_ extremity assumed to be host derived [26], [27]. This suggests that the endonuclease cleaves the host pre-mRNA directly after the first transcribed nucleotide, which is preferentially an adenosine. This difference with influenza virus could result from the fact that a similar mechanism is employed but the spatial disposition of the cap-binding and endonuclease domains is altered, or that the method of cap acquisition is mechanistically different in the two systems.

In this paper we present the atomic structures of the putative PB2 cap binding and 627 domains of THOV and the putative PA N-terminal endonuclease domains of THOV and DHOV. Despite very low sequence homologies, all domains have similar folds to those of the corresponding IAV polymerase domains. However, the critical active site residues in the putative cap binding and endonuclease domains are not conserved in THOV and DHOV and, contrary to the case of IAV polymerase, neither domain shows the expected *in vitro* biochemical activity. Our analysis suggests that Thogotoviruses are exceptional amongst orthomyxoviruses in that the lack of heterogeneous sequences at the 5′ end of the viral mRNA correlates with apparently degenerate cap-snatching domains in the polymerase. The origin and mechanism by which Thogotoviruses acquire the 5′ cap structure of the viral transcripts thus remains obscure.

## Materials and Methods

### Protein expression, purification and crystallization

THOV central PB2 domain (residues 323–486) was expressed as a 6xHis fusion in *E. coli* BL21 Codon Plus (DE3)-RIL (Stratagene) from a pProEXHTb expression vector (Invitrogen). THOV PB2 627 (543–701), THOV PA-Nter (1–169), THOV PA-Nter D86A (1–169) and DHOV PA-Nter (1–171) domains were expressed as 6xHis fusion protein from a pET-M11 vector (EMBL Heidelberg) using the same bacterial strain. Proteins were first purified by affinity chromatography using Ni-sepharose resin (GE-Healthcare), then cleaved with Tobacco Etch Virus (TEV) protease, further purified on a second Ni-sepharose column to remove His-tagged TEV and finally loaded on a Superdex 200 column (GE-Healthcare). After TEV cleavage, additional non-native N-terminal residues were left: THOV PB2 627 domain (GAM), THOV PA-Nter (GMGSGMA), DHOV PA-Nter (GA) and THOV central PB2 domain (GAMA).

THOV PB2 323–486 was concentrated to 9 mg/ml in a buffer containing 20 mM Tris pH 7.0, 200 mM NaCl and 5 mM β-mercaptoethanol. The best-diffracting crystals grew within 2 days at 20°C in a solution containing 100 mM Bis-Tris pH 6.0, 100 mM MgCl_2_ and 22% PEG3350. For data collection at 100 K, crystals were flash-frozen in liquid nitrogen with a solution containing mother liquor and 30% (v/v) glycerol. SeMet labelled PB2 323–486 was purified and crystallized in a similar way.

THOV PA 1–169 was concentrated up to 15 mg/mL in a buffer containing 100 mM NaCl, 20 mM Tris-HCl pH 8.0 and 10 mM β-mercaptoethanol. First crystals were obtained at 20°C in 20% PEG 4000, 0.1 M Mg acetate at pH 6–6.5 and the conditions were then refined to 0.1 M MgCl_2_, 0.1 M MES pH 6.5 and 22% PEG 4000. Crystals were cryo-protected with 20–25% glycerol before data collection. DHOV PA 1–171 protein was concentrated to 20 mg/mL in the same buffer as THOV PA-Nter domain. Crystals were grown at 20°C in 3.2 M Na formate and 0.1 M Hepes pH 7.5 and cryo-protected with 25% glycerol before flash-freezing. SeMet labelled THOV and DHOV PA-Nter domains were purified and crystallized in a similar way.

THOV PB2 543–701 was concentrated to 2.3 mg/mL in 250 mM NaCl, 50 mM Tris pH 8.0 and 2 mM DTT and crystals were grown in 0.1 M Hepes pH 7.0, 0.2 M ammonium sulphate and 22% PEG3350. Some of these crystals were further soaked in 5 mM HgCl_2_ for 1 h for use as a heavy atom derivative. Crystals were cryo-protected with 20% glycerol before flash-freezing in liquid nitrogen.

### Data collection and structure determination

Data collection and refinement statistic are given in Tables 1 and 2. Diffraction data were collected on various beamlines at the European Synchrotron Radiation Facility (ESRF, Grenoble, France), integrated and scaled with the XDS suite [28] and further processed using the CCP4i suite [29] with refinement using REFMAC5 [30] or PHENIX [31]. COOT [32] was used for graphical display and manual structure improvement. Structure figures were drawn with PYMOL [33]. Sequence alignments were made with ClustalW [34], with some manual adjustment, and drawn with ESPript [35].

**Table 1:** Data collection and refinement statistics.

	PA-Nter Thogoto	PA-Nter Thogoto	PA -Nter Dhori	PA -Nter Dhori
	SeMet Form 1	SeMet Form 2	Native	SeMet
**Data collection**				
Space group	*C*2	*C*2	*P*2_1_	*P*2_1_
Cell dimensions (Å)	a = 83.93, b = 77.23, c = 67.75	206.00, 79.16, 70.30	43.91, 87.45, 56.13	44.00, 87.42, 56.28
α, β, γ (°)	90, 123.27, 90	90, 97.23, 90	90, 112.98, 90	90, 112.99, 90
ESRF Beamline	BM14	ID29	ID29	ID14-4
Wavelength (Å)	0.9786	0.9792	0.9794	0.9791
Resolution* (Å)	50-2.7 0	50-2.77	50-1.30	50-1.70
Highest shell (Å)	2.70-2.80	2.77-2.87	1.30-1.35	1.70-1.78
*R*_sym_ * (%)	7.6 (68.4)	6.9 (29.7)	5.5 (37.0)	9.5 (39.7)
*I*/σ*I* *	13.5 (2.1)	9.6 (2.1)	14.8 (4.3)	10.4 (4.9)
Completeness* (%)	98.1 (97.9)	92.7 (51.4)	96.6 (94.2)	98.8 (99.3)
Redundancy*	3.8 (3.8)	2.1 (1.2)	3.41 (3.45)	2.52 (2.49)
**Refinement**				Not done
Resolution (Å)	50-2.7	50-2.77	50-1.30	
No. refl. work/free	9408/471	25357/1354	87879/4640	
*R*_work_ *	0.199 (0.260)	0.229 (0.324)	0.173 (0.230)	
*R*_free_ *	0.276 (0.341)	0.279 (0.389)	0.220 (0.306)	
Overall No. atoms	2601	7451	3390	
Protein	2601 (2 molecules)	7451 (6 molecules)	2906 (2 molecules)	
Water	-	-	472+12 glycerol	
Overall *B*-factor (Å^2^)	71.0	47.2	13.3	
Protein	71.0	47.2	11.5	
Water	-	-	24.5	
R.M.S. deviations				
Bond lengths (Å)	0.004	0.005	0.020	
Bond angles (°)	0.827	0.947	1.928	
Ramachandran plot**				
Favoured %	97.1	98.4	98.2	
Allowed %	100.0	99.1	99.5	

Values in parentheses are for highest resolution shell.

http://molprobity.biochem.duke.edu/index.php.

**Table 2:** Data collection and refinement statistics (continued).

	PB2 ‘cap domain’	PB2 ‘cap domain’	PB2 ‘cap domain’	PB2 627 domain	PB2 627 domain
	Native Form 1	SeMet Form 1	Native Form 2	Native	Hg derivative
Data collection					
Space group	*I*4	*I*4	*P*42_1_2	*P*4_3_2_1_2	*P*4_3_2_1_2
Cell dimensions (Å)	a = 109.6, b = 109.6, c = 39.1	109.1, 109.1, 40.4	101.44, 101.44, 106.02	65.48, 65.48, 74.78	65.12, 65.12, 73.38
α, β, γ (°)	90, 90, 90	90, 90, 90	90, 90, 90	90, 90, 90	90, 90, 90
ESRF Beamline	ID23-2	ID29	ID23-2	ID23-2	ID23-2
Wavelength (Å)	0.8726	0.9792	0.8726	0.8726	0.8726
Resolution (Å)	50-1.80	50-2.70	50.0-3.0	50-2.40	50-2.80
Highest shell (Å)	1.80-1.85	2.70-2.80	3.00-3.11	2.40-2.50	2.80-2.93
Rsym* (%)	6.5 (64.5)	4.4 (58.5)	22.2 (95.4)	8.4 (56.1)	10.7 (63.9)
I/σI*	16.5 (2.4)	12.2 (1.9)	12.9 (3.8)	11.1 (2.6)	11.2 (3.1)
Completeness (%)	99.7 (99.6)	97.6 (97.7)	99.9 (99.9)	97.4 (98.7)	99.9 (100)
Redundancy	5.2 (3.7)	2.1 (2.1)	10.2 (10.4)	3.95 (4.01)	4.45 (4.51)
**Refinement**		Not done			Not done
Resolution (Å)	50-1.8		50-3.0	50-2.40	
No. refl. work/free	20631/1115		11056/554	6288/316	
*R*_work_ *	0.172 (0.256)		0.200 (0.263)	0.208 (0.308)	
*R*_free_ *	0.192 (0.332)		0.216 (0.289)	0.239 (0.499)	
Overall No. atoms	1525		2635	1090	
Protein	1337		2635	1073	
Water	188		-	17	
Overall B-factor (Å^2^)	29.1		34.8	50.4	
Protein	27.6		34.8	50.6	
Water	40.3		-	39.6	
R.M.S. deviations					
Bond lengths (Å)	0.007		0.003	0.006	
Bond angles (°)	1.204		0.846	1.054	
Ramachandran plot**					
Favoured %	97.5		96.9	97.7	
Allowed %	100.0		99.7	99.2	

Values in parentheses are for highest resolution shell.

http://molprobity.biochem.duke.edu/index.php.

The best crystals of THOV PB2 323–486 belong to the space group *I*_4_ with one molecule per asymmetric unit and diffracted to 1.8 Å resolution. A second crystal form was of space-group *P*42_1_2 with two molecules per asymmetric unit and diffracted to 3 Å resolution. The structure was solved by the single anomalous dispersion (SAD) method using 2.7 Å resolution data collected on SeMet labelled crystals at the Se K-edge. Three selenium sites were identified, refined and used for phasing with autoSHARP [36]. After density modification, clear electron density covering the entire molecule was obtained. The experimental phases were transferred to the nearly isomorphous 1.8 Å resolution native data set and most of the model was built automatically using ARP-wARP [37]. The model was refined to a final R-free/R-work of 19.2/17.2%. The THOV putative cap-binding domain model contains residues 326–485. The second crystal form was refined to a final R-free/R-work of 21.6/20.0%.

Crystals of DHOV PA-Nter were of space-group *P*2_1_ with two molecules in the asymmetric unit and diffracted to 1.3 Å resolution. The structure was solved by the SAD method, as described above, using 1.7 Å resolution data from SeMet labelled crystals measured at the Se K-edge. The native structure was refined to a final R-free/R-work of 22.0/17.3%. The DHOV PA-Nter model contains residues 1–168.

Two crystal forms of THOV PA-Nter were obtained, both of *C*2 space-group and either two (2.7 Å resolution) or six (2.77 Å resolution) molecules in the asymmetric unit. The structure could not be solved by molecular replacement using PA-Nter due to the low sequence homology. Attempts to *de novo* phase by Se-Met labelling failed due to the too weak signal from the low number of methionines and even after making point mutations to introduce more methionines. The structure was finally solved by molecular replacement using the structure of DHOV PA-Nter using PHASER [38]. The small *C*2 cell model was refined to an R-free/R-work of 27.6/19.9%. The large *C*2 cell model was refined to an R-free/R-work of 27.1/21.0%. The THOV PA-Nter model contains residues 2–166.

Crystals of the THOV PB2 627 domain were of space-group *P*4_3_2_1_2 with one molecule in the asymmetric unit and diffracted to 2.4 Å resolution. The structure was solved by the SAD method using 2.8 Å resolution data from HgCl_2_ soaked crystals. One Hg site was sufficient to solve the structure. The native structure was refined to a final R-free/R-work of 23.9/20.8%. The THOV 627 domain model contains residues 547–681.

### Cap-binding assay

IAV cap-binding domain (H3N2, residues 318–483, [15]) and THOV putative cap binding domain (323–486), purified as described above, were loaded on m^7^GTP sepharose resin (GE-Healthcare), bound for 3 h at 4°C, washed extensively with buffer containing 200 mM NaCl, 50 mM Tris-HCl pH 8.0 and 2 mM DTT, and eluted in the presence of 1 mM m^7^GTP. Input, washed and eluted fractions were analyzed on SDS-PAGE 15% acrylamide gel stained with Coomassie blue.

### Endonuclease assay

The nuclease activity of IAV PA-Nter domain (H3N2 PA residues 1–209, [16]) and putative THOV endonuclease domain (PA 1–169) was tested by incubating proteins with various RNA substrates: *Alu* domain RNA (110 nucleotides highly structured RNA), short flu panhandle RNA (36 nucleotides structured RNA) and poly(U) RNA (51 nucleotides unstructured RNA) as described [16], [39]. After 30 min incubation at 30°C or 37°C, samples were analyzed on 8 M urea 15% acrylamide gel stained with methylene blue.

### Biophysical assays

Thermal shift assays were performed with 10 µM THOV or DHOV PA-Nter in 20 mM Tris-HCl pH 8.0, 100 mM NaCl and 10 mM β-mercaptoethanol and a 5× dilution of SYPRO Orange dye (Invitrogen) as described [40]. The dye was excited at 490 nm and the emission light was recorded at 575 nm while the temperature was increased by increments of 1°C per minute from 25 to 75°C.

Isothermal titration calorimetry (ITC) was performed at 25°C with an ITC200 micro-calorimeter (Microcal) and consisted of 25 injections of 1.5 µL m^7^GTP ligand in a cell containing PB2 domains. IAV and THOV central PB2 domains concentrated to about 75 µM were extensively dialysed in buffer containing 200 mM NaCl, 50 mM Tris pH 7.5 and 5 mM β-mercaptoethanol before measurements. m^7^GTP was injected at 0.6 mM for the IAV and 4 mM for the THOV experiments. Data were analysed with Origin7 soft (Microcal).

### THOV minireplicon assay

The cDNAs encoding the the viral polymerase subunits and NP of THOV were cloned into eukaryotic expression vector pCAGGS. For detection of the recombinant proteins that contain single amino acid exchanges in PB2(R344A) and PA(D86A) by Western blot, cDNAs encoding wild-type and mutant proteins were fused to a C-terminal Flag-tag for PB2 or HA-tag for PA, respectively.

Minireplicon assays were performed by transfecting 293T cells in 12 well format with 250 ng of pCAGGS-expression plasmids encoding PB2, PB1 and PA, 500 ng of NP as well as 100 ng of the viral minigenome construct pHH21-vNP-FF-Luc, containing firefly luciferase (FF-luc) cDNA in antisense orientation flanked by the noncoding regions of THOV segment 5 as described [41]. In addition, the transfection mixture contained 20 ng of pRL-SV40 constitutively expressing Renilla luciferase under the control of the SV40 promoter. At 24 h post transfection, cells were lysed and luciferase activities were determined using dual luciferase reporter assay (Promega). Relative polymerase activity was calculated as the ratio of firefly luciferase to Renilla luciferase luminescence.

## Results

### Putative endonuclease domain of Thogoto virus PA

Based on a sequence alignment with the IAV PA, a first construct (residues 1 to 185) was designed for the N-terminal domain of THOV PA subunit and the corresponding protein was expressed in bacteria and purified. Limited proteolysis with papain followed by mass spectrometry revealed a shorter, protease resistant fragment. The corresponding construct (residues 1 to 169) was cloned, expressed, purified and crystallized. The structure could not be solved by molecular replacement using that of IAV PA-Nter [16] due to low sequence homology. Furthermore, attempts to *de novo* phase by seleno-methionine labelling failed due to the too weak signal from the low number of methionines. To overcome this problem, we produced the homologous domain from DHOV PA (40% identity between full-length THOV and DHOV PA, 29% for the N-terminal part), which readily crystallized and the structure was solved by the Single wavelength Anomalous Dispersion (SAD) method using seleno-methionine labelled protein. The resultant model was then used to solve the THOV PA-Nter structure by molecular replacement. Crystallographic details are given in Table 1.

**Figure 1:**
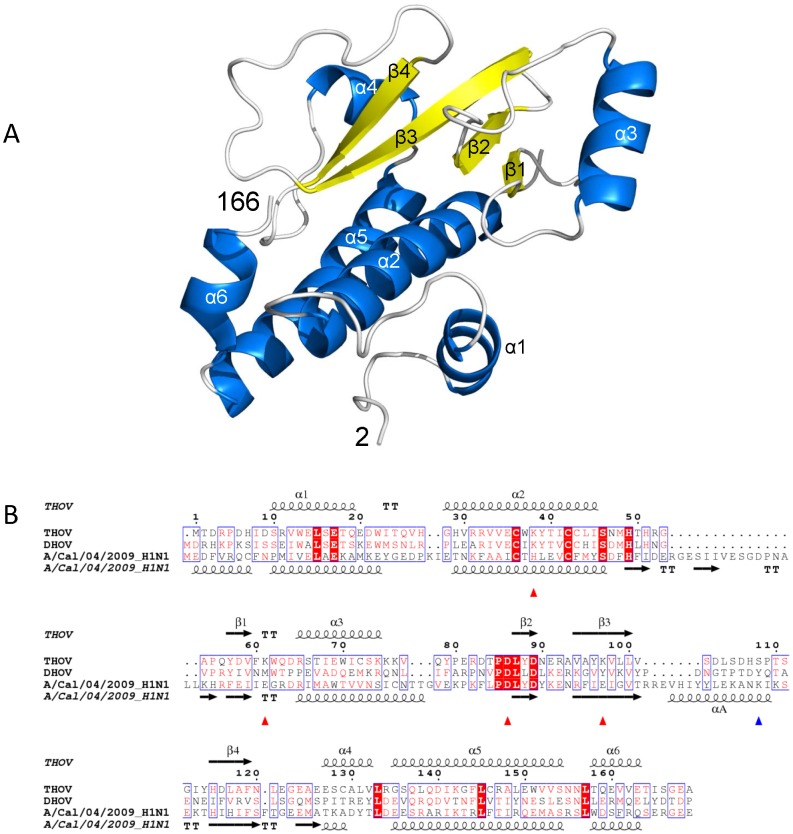
Structure of THOV PA-Nter domain. A. Ribbon diagram showing the structure of the THOV PA-Nter domain. Helices are in cyan and beta strands in yellow and labeled according to the alignment in (B). B. Structure based sequence alignment of THOV, DHOV and IAV PA-Nter domains showing secondary structure elements for THOV and INFA. Red triangles indicate cation binding residues in IAV (His41, Glu80, Asp108 and Glu119). A blue triangle indicates the catalytic lysine (Lys134) in IAV which is on helix αA in INFA that does not exist in THOV and DHOV.

The structure of THOV PA-Nter is shown in Figure 1A, together with a structure based sequence alignment of various orthomyxovirus PA-Nter domains (Figure 1B, a more complete alignment is shown in Figure S1 in File S1). THOV PA-Nter comprises four principle β-strands surrounded by six α-helices. Comparison of the two independent molecules in the asymmetric unit of crystal form 1 (Table 1) shows that the region 72–89, including helix α3 and the following loop, is in two very distinct orientations (Figure S2A in File S1). In chain A, this element is positioned as in DHOV and IAV (see below) whereas in chain B it is dissociated from the body of the protein, rotated by 90° and inserted between two neighbouring molecules in the crystal (Figure S2B in File S1). Interestingly, this asymmetry is also observed in crystal form 2 of THOV PA-Nter (Table 1) in which there are six molecules in the asymmetric unit. These are arranged as three asymmetric dimers, each similar to that observed in form 1, but for the chain in which the 72–89 element is dissociated from the body of the protein, the electron density for the element is lacking, presumably due to mobility. The significance of this observation is not clear but it indicates a certain plasticity of the THOV PA-Nter structure, not observed in the DHOV structure and correlates with a lower thermal stability of the THOV domain (see below).

**Figure 2:**
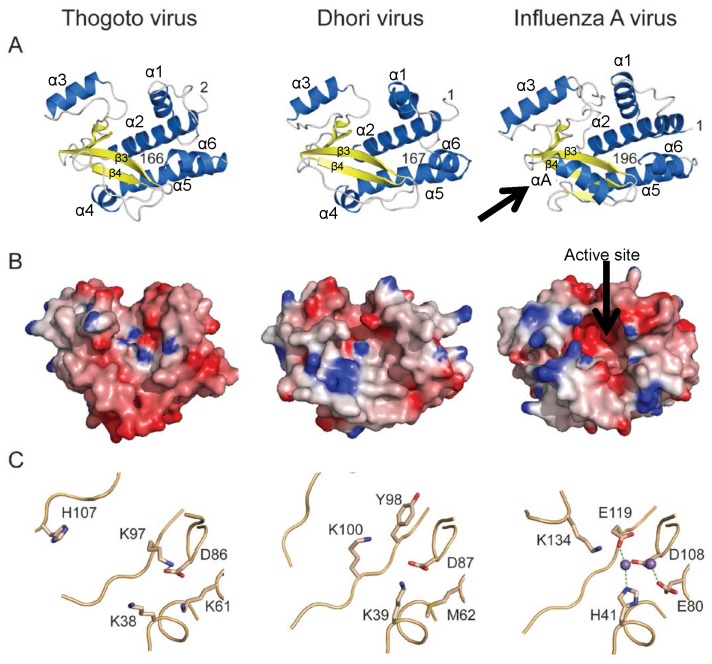
Structural comparison of THOV, DHOV and IAV PA-Nter domains. A. Comparative structures of THOV, DHOV and IAV PA-Nter domains, in the same orientation after superposition, with coloring and secondary structure elements as in Figure 1A. Note helix αA (arrowed in right panel), which carries the catalytic lysine in IAV, is replaced by an irregular strand in THOV and DHOV. The root-mean-square deviation (RMSD) between THOV and DHOV is 1.47 Å for 142/169 aligned Cα<3.8 Å apart, and between THOV and IAV is 1.62 Å for 122 aligned Cα<3.8 Å apart. B. Electrostatic surfaces for the three domains (red, negatively charged; blue, positively charged). The IAV active site (right, arrowed) is negatively charged with a rim of positive charge, whereas THOV and DHOV have more positively charged residues within the active site. C. Comparison of residues in THOV and DHOV equivalent to the functionally important active site residues of IAV including the two bound cations. Only Asp86, Asp87 and Asp108 are conserved in THOV, DHOV and IAV, respectively.

Figure 2 shows the comparison between the PA-Nter domains of THOV, DHOV and IAV. The three domains clearly have the same fold, and THOV and DHOV PA-Nter are very similar (Figure 2A). However, IAV PA-Nter has some significant differences from the THOV and DHOV structures. First, there is an extra loop of 16 residues following helix α2, known to be flexible [22], which is only conserved in influenza B PA but not in other orthomyxoviruses (Figure S1 in File S1). Second, there are important differences in the putative endonuclease site. IAV PA-Nter has a negatively-charged cavity which binds two octahedrally co-ordinated divalent cations with the side chains of His41, Glu80, Asp108 and Glu119 and the main chain of Ile120 [16], [22], [39]. For THOV and DHOV PA-Nter the putative active site is more like an extended groove (Figure 2B) and furthermore, is positively charged. This is explained by the non-conservation of the key active site residues identified in influenza endonuclease. Indeed of the cation binding residues, only an equivalent of influenza Asp108 (PD motif) is found in THOV and DHOV PA-Nter, respectively Asp-86 and Asp-87 (Figure 1B and 2C). According to the structure based alignment (Figure 1B), His41 in IAV is equivalent to Lys38 in THOV (Lys39 in DHOV), Glu80 to Lys61 in THOV (Met62 in DHOV) and Glu119 is substituted by Lys97 in THOV (Tyr98 in DHOV). Moreover, there is no apparent correspondence to the catalytic Lys134 of influenza PA-Nter, since the entire helix bearing this residue in IAV PA-Nter (αA) is replaced in THOV and DHOV by an extended, but irregular connection between strands β3 and β4 (Figure 1B, 2A). The extra positive charge in the putative active sites of THOV and DHOV PA-Nter arises from the replacement of acidic residues and the introduction of extra lysines (Lys38, Lys61 and Lys97 for THOV and Lys39 and Lys100 for DHOV PA-Nter). Of all the putative active site residues, only two are conserved between THOV and DHOV (Lys38/39 and Asp86/87). However the PA sequence from JOSV [9] reveals further divergence since neither of these residues (not even the aspartate of the PD motif, which becomes HD) is conserved in JOSV PA-Nter (Figure S1 in File S1). For the catalytic lysine (Lys134 in IAV), conservation is less clear, since its sequential position is not necessarily maintained in PD/DxK nucleases [12]. All these observations raise the question of whether the THOV, DHOV and JOSV PA-Nter domains maintain endonuclease activity. On the other hand, the PA-Nter sequences of infectious salmon anaemia and Quaranfil virus conserve the key active site residues (Figure S1 in File S1) suggesting that they have an active influenza-like endonuclease.

**Figure 3:**
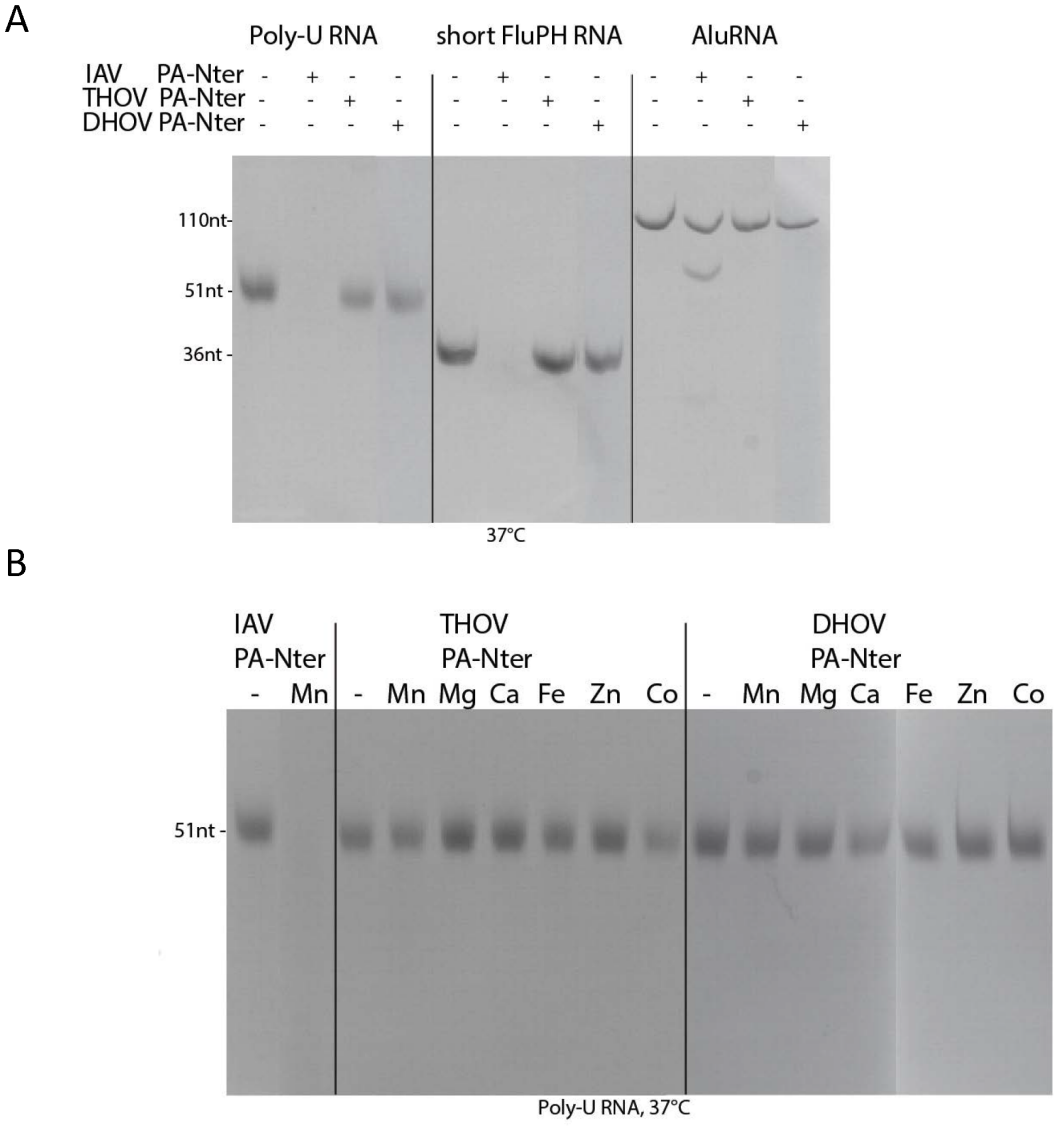
Functional comparison of THOV, DHOV and IAV PA-Nter domains. A. Denaturing urea gel analysis of an endonuclease assay in which IAV, DHOV and THOV PA-Nter domains were incubated at 37°C for 30 minutes in the presence of 1 mM MnCl_2_ with U-rich RNA (left lanes), short Flu panhandle RNA (middle lanes) or *Alu* domain RNA (right lanes) as described [16]. IAV endonuclease readily degrades the ssRNA and partially the structured *Alu* domain RNA, but THOV and DHOV PA-Nter are inactive under these conditions. B. Similar assay with THOV and DHOV PA-Nter and U-rich RNA in the presence or absence of various divalent ions (1 mM MnCl_2_, MgCl_2_, CaCl_2_, FeCl_2_, ZnCl_2_ or CoCl_2_). IAV endonuclease degrades the RNA in the presence of manganese but THOV PA-Nter does not degrade the RNA with any cation.

In previous work we have shown that the isolated PA-Nter domain of IAV is thermally stabilised by binding certain divalent cations and has manganese-dependent endonuclease activity against ssRNA [16], [39]. We therefore tested whether the equivalent domain of THOV has similar biochemical properties. Using the Thermofluor assay to measure apparent melting temperature (Tm), we found that in the absence of any divalent cations, the Tm of THOV and DHOV PA-Nter domains were respectively 41 and 53°C (Figure S3 in File S1) compared with 44°C for IAV [16]. Upon addition of Mn^2+^, Ca^2+^ or Mg ^2+^ ions, the IAV domain is thermally stabilised by 13, 8 or 6°C respectively, consistent with metal binding [16]. For the DHOV domain, which is already more stable, addition of these ions has little effect, giving no indication of cation binding (Figure S3 in File S1). Surprisingly, for the THOV domain, addition of Mn^2+^ or Mg^2+^ ions destabilises the protein by 3–6°C (Figure S3 in File S1). Interestingly, the significant difference in thermal stability behaviour of the THOV and DHOV domains correlates with the enhanced plasticity and much poorer diffracting crystals of THOV PA-Nter, as described above. Finally, neither THOV nor DHOV PA-Nter show any nuclease activity against various RNAs under conditions where IAV PA-Nter does (Figure 3AB). Our structural and biochemical results therefore strongly suggest that THOV and DHOV PA-Nter domains neither bind divalent cations nor are active endonucleases.

Finally, in the THOV PA-Nter domain, a D86A mutation was made in the PD motif that is conserved in most orthomyxovirus PA subunits, except JOSV (Figure S1 in File S1). For IAV, the equivalent residue (Asp108) binds both of the divalent metal ions in the endonuclease active site and its mutation to alanine abolishes nuclease activity *in vitro* [39] and viral transcription, but not replication, in minireplicon assays [17], [42]. We found that the D86A mutated domain was still well-expressed, but became totally insoluble (not shown). This destabilisation through misfolding is likely due to electrostatic imbalance since D86 is the only negatively charged residue amongst three positively charged residues in the putative active site (Figure 2C) and in any case, as shown above, the domain is thermally labile. The D86A mutant in PA was also tested in a minireplicon assay, in which THOV polymerase activity is detected via a firefly luciferase encoding viral minigenome. Not unexpectedly the mutation was found to abolish polymerase activity (not shown) suggesting that a well-folded PA-Nter domain is required but not shedding further insight on its role.

### ‘Cap-binding’ domain of PB2

Sequence alignments were used to define a putative cap-binding domain in THOV PB2 subunit, even though the sequence identity of this region of PB2 compared to IAV PB2 is only about 8% (Figure S4 in File S1). A construct comprising residues 323–486 could be expressed, crystallised in two different forms, and its structure determined at 1.8 Å resolution (Table 2). Once again, the low sequence homology with the corresponding IAV domain necessitated *de novo* structure solution by selenium SAD, rather than by molecular replacement.

**Figure 4:**
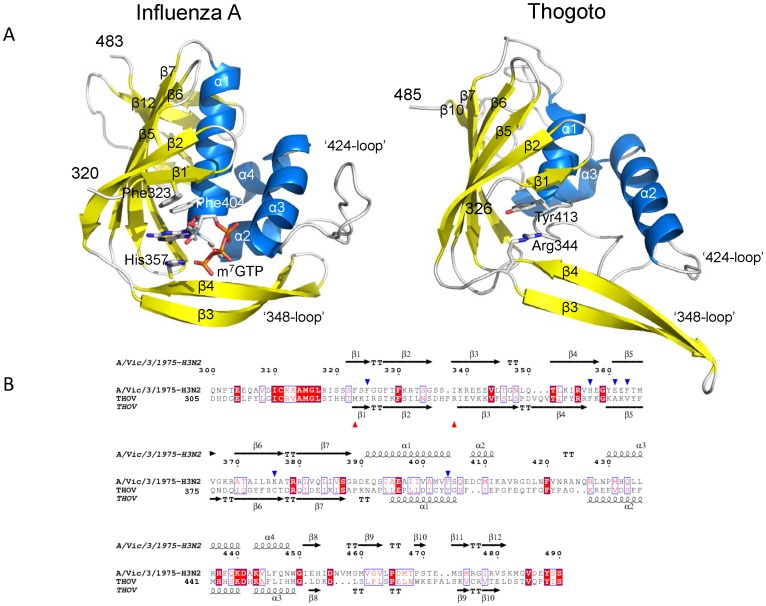
Structural comparison of THOV and IAV central PB2 domains. A. Ribbon diagram comparing the structure of the IAV (left) and THOV (right) central PB2 domains, in the same orientation after superposition. Helices are in cyan and beta strands in yellow and labeled according to the alignment in (B). In the IAV, the bound cap analogue m^7^GTP is depicted together with three aromatic residues (Phe323, His357 and Phe404) involved in ligand binding. In the THOV domain, Arg344, which occupies the position equivalent to that of the m^7^G base, and Tyr413 (equivalent to Phe404) are shown. Comparison of the THOV and IAV domains using PDBeFOLD (http://www.ebi.ac.uk/msd-srv/ssm/) gives an RMSD = 3.0 Å for 133/160 matched Cα and Z = 6.1. B. Structure based sequence alignment of THOV and IAV central PB2 domains showing secondary structure elements. Blue triangles indicate the key residues involved in ligand binding in the case of IAV (Phe323, His357, Glu361, Phe363, Lys376 and Phe404). Red triangles indicate THOV residues that would clash with bound m^7^GTP (Met328 and Arg344, see Figure 5A).

A comparison of the IAV and THOV central PB2 domains is shown in Figure 4A. Despite the low sequence identity the THOV domain is clearly structurally homologous to that of IAV as indicated by the close correspondence of secondary structure elements shown in the structure based alignment (Figure 4B). However, apart from the different length and orientation of the helices, there are two other notable differences in the path of the polypeptide. Firstly, the so called ‘348-loop’ [15] formed by the β3–β4 hairpin (Figure 4A) is longer in THOV and its extremity orientates away from the body of the domain towards the solvent, whereas in IAV the tip packs against bends the body of the molecule (Figure 4A). This is apparently not an artefact of crystal packing as a very similar conformation of this beta hairpin is observed in the second crystal form. Secondly, the prominent, solvent exposed ‘424-loop’ of the IAV cap-binding domain is absent due to truncation in THOV. The integrity of this loop was shown to be required for cap-dependent transcription in IAV [15].

**Figure 5:**
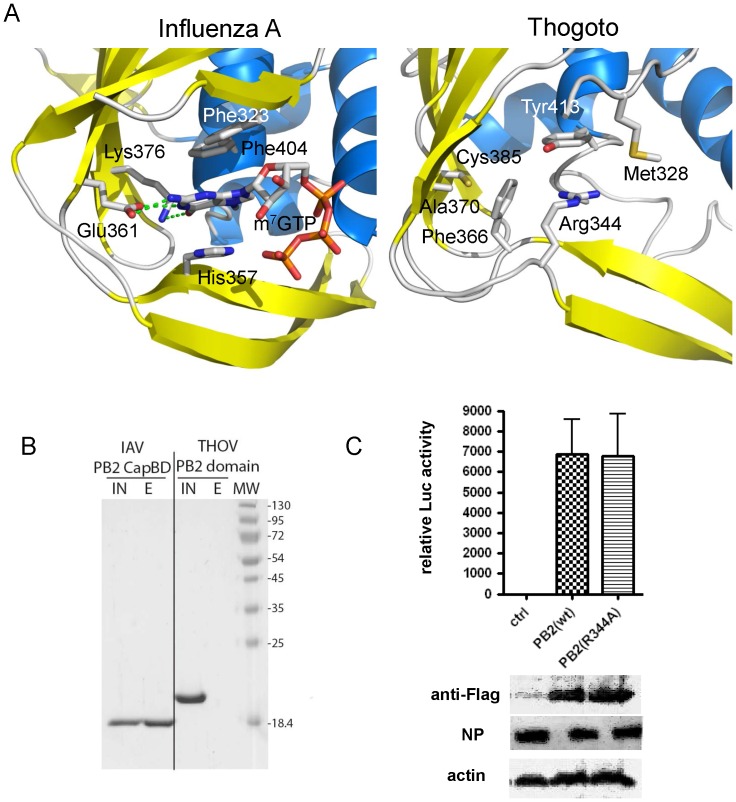
Functional comparison of THOV and IAV central PB2 domains. A. Comparison of m^7^GTP binding site and key interacting residues in IAV (left) and structurally equivalent residues in THOV (right) central PB2 domain. Colours as in (Figure 4A). Guanine base specific contacting residues in IAV (Glu361 and Lys376) are non-conservatively substituted by Ala370 and Cys385 in THOV, respectively and the aromatic residue Phe366 is differently orientated in THOV from His357 in IAV. In the THOV domain Arg344 occupies the position equivalent to that of the m^7^G base and Met328 that of the ribose and only aromatic residue Tyr413 (equivalent to Phe404 in IAV) is conservatively substituted. B. Cap-binding assay with THOV and IAV central PB2 domains. SDS PAGE analysis of the results of elution of IAV (IAV-capBD) and putative THOV cap binding domains (THOV-capBD) after binding on 7-methyl-GTP sepharose resin at 4°C. IAV-capBD (left lanes) was used as a positive control and binds the resin, whereas THOV-capBD (right lanes) does not. (IN) and (E) indicate input and eluted fractions respectively. Molecular weight markers (MW, kDa)) are shown in the right most lane. C. Minireplicon assay of the PB2 R344A mutant. THOV polymerase activity was determined in a reconstituted minireplicon system by transfecting 293T cells for 36 h with expression plasmids coding for a FF-Luc-encoding viral minigenome and viral proteins PB2, PB1, PA and NP. The PB2 subunit was transfected as Flag-tagged PB2(wt)(250 ng) or PB2(R344A)(350 ng) constructs to allow detection of the recombinant proteins by Western blot using specific antibodies. Firefly luciferase activities were normalized to Renilla. Mean values from two independent experiments with duplicates are shown. The error bars represent standard deviation.

The most significant differences are in the putative ligand binding site, which in IAV binds the cap structure [15]. In IAV, the 7-methylated guanosine (m^7^G) is sandwiched between aromatic residues His357 and Phe404, with Glu361 and Lys376 making base-specific interactions. The THOV domain conserves very few of these features which are characteristic of a cap binding site (Figure 5A). Whereas in THOV, Tyr413 is similarly placed to Phe404, there is no equivalent of His 357 for the other side of the aromatic sandwich. Indeed, the side chain of Phe366 in THOV, which misleadingly aligns with His357 of IAV (Figure 4B), is orientated not parallel to Tyr413, but nearly perpendicular and in fact occupies the space equivalent to the functionally important side chain of Glu361 in IAV (Figure 5A). Furthermore, there are no polar residues in THOV that could specifically hydrogen bond to the guanosine base, since Glu361 and Lys376 in IAV are non-conservatively replaced by respectively Ala370 and Cys385 (Figure 5A) (Tyr and Gly in DHOV, Figure S4 in File S1). Finally, in all three crystallographically independent examples of the THOV domain, the equivalent position of the m^7^G itself is occupied by the side-chain of Arg344, with its guanidinium group stacking with the side-chain and making two hydrogen bonds to the main-chain carbonyl oxygen of Tyr413. Met328 in THOV would also clash with the position of the ribose, whereas the equivalent residue in IAV is Phe323 which packs against the ribose (Figure 5A). Thus the significantly different nature and arrangement of the residues within the putative active site of THOV would appear to preclude any possible binding of m^7^G in a similar manner to that observed in IAV. Furthermore, given the high resolution, well-ordered structure of the THOV domain, which is the same for all three crystallographically independent copies observed, it is extremely unlikely that the putative active site region could be repacked through induced fit to allow m7G binding. The structure based sequence alignment (Figure S4 in File S1) suggests that THOV, DHOV and JOSV PB2 domains share the same structure and distinctive features (e.g. Arg344, Phe366 and Tyr 413 are conserved between them).

To test whether or not the central THOV PB2 domain binds cap we used an m^7^G Sepharose binding assay. As shown in Figure 5B, the THOV domain is not retained by m^7^G Sepharose under conditions where IAV cap-binding domain is and, similarly, no binding of m^7^GTP could be detected by isothermal calorimetry experiments under conditions where IAV cap-binding domain shows clear binding with a K_d_ of 2.7 µM (Figure S5 in File S1). We also made the point mutation R344A in the isolated THOV central PB2 domain, since we thought that this residue, which stacks on Tyr413 and is positioned where the m^7^G is bound in the IAV domain, might be auto-inhibiting cap-binding. The recombinant, mutated domain purified as wild-type but still did not bind m^7^G Sepharose (not shown). Finally we made this same mutation in the context of the complete PB2 subunit and tested the effect in the THOV mini-replicon system. We found that the PB2 R344A mutation had no effect on the expression level of the PB2 subunit (detected via a C-terminal Flag-tag by Western blot) nor on polymerase activity (detected by luciferase activity) (Figure 5C).

### ‘627’ domain of PB2

**Figure 6:**
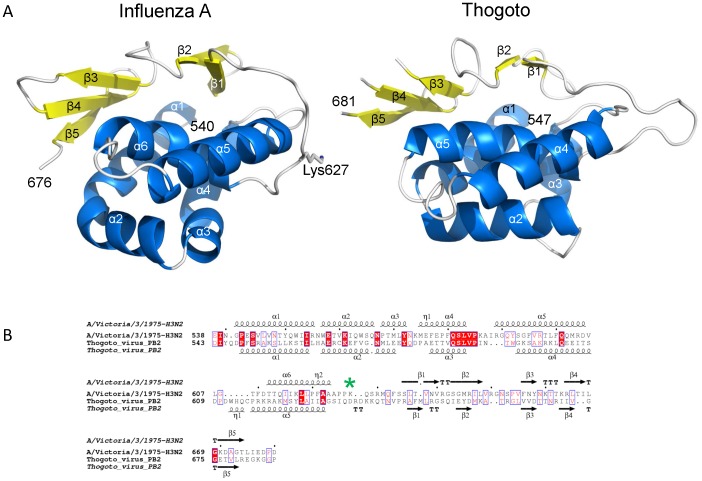
Structural comparison of THOV and IAV PB2 627-domain. A. Ribbon diagram comparing the structure of the IAV (left) and THOV (right) PB2 627 domains, in the same orientation after superposition. Helices are in cyan and beta strands in yellow and labeled according to the alignment in (B). The species specific residue Lys627 is shown for IAV; the corresponding loop in THOV is two residues longer. Comparison of the THOV and IAV (PDB 2VY7) domains using PDBeFOLD gives an RMSD = 2.6 Å for 114/154 matched Cα and Z = 5.0. B. Structure based sequence alignment of THOV and IAV PB2 627 domains showing secondary structure elements. Lys627 in the IAV domain is highlight with a green asterisk.

To explore further the structural and functional similarity between THOV and influenza polymerase domains, we determined the structure of the so-called 627-domain [19] of THOV by crystallising a construct comprising residues 543–701 of THOV PB2. The structure was solved *de novo* using anomalous scattering from a mercury derivative and refined to 2.4 Å resolution (Table 2). Again, despite little significant sequence identity (∼8%) between THOV and IAV in this region of PB2 (although the motif QSLVP at the end of helix α3/4 is conserved), the domains clearly have the same fold (Figure 6A, B). Interestingly the loop which in IAV carries the host-specific residue Lys/Glu627 [19] is extended by two residues in THOV. A large region of the IAV 627-domain surface is positively charged (Figure S6 in File S1), consistent with its ability to bind RNA [43]. The corresponding electrostatic surface of THOV 627-domain also has positive patches but less uniformly (Figure S6 in File S1). The structure-based multiple sequence alignment of this region of PB2 strongly suggests that DHOV, Jos and Quaranfil have a similar three dimensional structure to THOV, although Quaranfil is more diverged (Figure S7 in File S1).

## Discussion

Thogoto and Dhori viruses are tick borne orthomyxoviruses with structural and genetic properties similar to influenza viruses but also some significant differences. It is currently thought that all orthomyxoviruses perform transcription in the nucleus using short capped primers derived by cap-snatching. Consistent with this, THOV, DHOV and IAV all depend on host cell RNA polymerase II activity for synthesis of viral mRNAs [44], thus maintaining a supply of nascent pre-mRNAs to be pirated. Whereas IAV mRNAs have heterogeneous host derived sequences of length 10–13 nucleotides from the cap [11], [45], [46], THOV mRNAs have 5′ ends that are uniform in length and exact copies of the vRNA templates, starting preferentially with an A at the cap-proximal position [25]–[27]. Using purified viral cores it has also been shown that endonuclease activity of THOV polymerase depends on the presence of the 5′ and 3′ ends of the vRNA [25]. Recently, the influenza polymerase PB2 cap-binding and PA endonuclease domains have been identified and the recombinantly expressed domains show the corresponding biochemical activity [15], [16]. We therefore set out to determine whether domains with similar activities existed in THOV polymerase and whether a structural explanation could be found for the observed differences in the cap-snatching mechanism described for THOV and IAV.

THOV and IAV polymerases are clearly homologous with 22% identity averaged over the three subunits. However the homology is not uniform, with the highest being in the C-terminal region of PA, the PB1 subunit and the N-terminal region of PB2. In other regions, particularly the C-terminal half of PB2, the relatively low identity (∼8%) makes alignments uncertain, without structural information. In addition, the PA and PB1 subunits are considerably shorter (respectively 622 and 710 compared to 736 and 757 for IAV), whereas the THOV PB2 subunit is slightly longer (769 compared to 759 in IAV). Nevertheless we were able to express, purify and determine the crystal structure of the three THOV polymerase domains corresponding to the PA endonuclease and PB2 cap-binding and 627-domains, previously structurally and functionally characterised for IAV polymerase (reviewed in [18]). The THOV domain structures determined confirm the fundamental structural homology between THOV and IAV polymerase domains, but in each case the sequence divergence, which translates into a relatively large RMSD of C-alpha positions, necessitated *de novo* structure determination rather than molecular replacement. The most surprising observation was that neither the putative PA endonuclease nor the putative PB2 cap-binding domains of THOV have the corresponding *in vitro* biochemical activities, respectively divalent metal ion binding, ssRNA cleavage and cap-binding, as found for the isolated IAV domains. This correlates with non-conservative substitution of functionally critical divalent cation binding and catalytic residues that are fully conserved in the endonuclease active sites of all influenza A, B and C polymerases and similarly for key residues that bind the methylated guanine in the cap-binding domain. In the case of the PA-Nter, it is striking that even the more closely related THOV, DHOV and JOSV domains show no fully conserved residues in the putative active site. In the case of the putative cap-binding domain of THOV PB2, the ligand binding site has been remodelled to be unfavourable to cap-binding, no longer maintaining either the aromatic sandwich or the hydrophilic residues that could specifically recognise m^7^G. This we are tempted to conclude that neither domain is active in cap-snatching.

Previous work on THOV replication has shown that THOV mRNAs are capped but lack heterogeneous sequences at the 5′ end [26], [27] and this is also reported for a third Thogotovirus, JOSV [9]. This observation, coupled with the fact that THOV replication depends on on-going Pol II transcription [44], the assumed lack of a virally encoded capping enzyme and the apparent demonstration of capped RNA cleavage by purified viral cores [25] suggested that THOV primes transcription by cap-snatching. How to reconcile these conclusions with the apparent structural degeneration and lack of *in vitro* activity of the putative THOV polymerase cap-snatching domains? The overall sequence homology of THOV and IAV polymerase and structural similarity of the three domains described here strongly suggests that the proteins have a similar overall three-dimensional architecture. For IAV, the physical separation of the PB2 cap-binding and PA endonuclease domains and/or the path of the RNA presumably determine that the cleavage occurs at 10–13 nucleotides from the cap. THOV, which apparently cleaves directly after the cap, would seemingly have two major possibilities. First, the two domains could function as in IAV but are correspondingly physically closer, or the path of the RNA is more direct. This could be correlated with the smaller size of the PA and PB1 subunits (by respectively 88 and 47 residues), notably the shorter linker between the small N- and large C-terminal domains of THOV PA. The fact that the two THOV cap-snatching domains are apparently inactive *in vitro* could then be attributed to the need for activation by other parts of the polymerase or the vRNA as suggested previously [25]. This explanation is plausible, but it would be extremely surprising that the domains were so diverged from those of IAV if they basically functioned in the same way. The second possibility is that THOV polymerase requires a fundamental change in domain functions to perform snatching adjacent to the cap. A possible explanation could be that the PB2 cap-binding domain is no longer functionally required (being too far from the cleavage site) and is primarily maintained for the overall architectural integrity of the PB2 subunit. This would explain the degeneration of the ligand binding site although other possible functions of the domain, such as RNA binding could be conserved. Instead the PA-Nter domain could combine both activities and bind host m^7^GpppA_m_NN…. as substrate and cleave after A_m_, more akin to a decapping activity. This is plausible as the IAV endonuclease active site cleft is presumed to bind a minimum of about 5 nucleotides [46], [47]. Accordingly, it is difficult to imagine how distinct cap-binding and endonuclease domains could simultaneously bind to such a short substrate. It would also be consistent with a redesign of the THOV PA-Nter active site since it would need to bind the triphosphate of the m^7^Gppp moiety and hence might be expected to be more basic, as indeed observed (see above). However if the THOV, DHOV and JOSV PA-Nter domains really did have this very specific and novel activity one would expect them to show much more conservation than observed e.g. metal binding and catalytic residues. A further possibility is that both THOV domains are indeed inactive and that cap binding and cleavage are both performed directly in the polymerase active site. Finally it is possible that THOV does not actually do cap-snatching at all but that host encoded activities generate short capped RNAs. One scenario could be that viral transcription is primed with a single A and then capping is performed post-initiation by the cellular capping enzymes. Another scenario would be that free m^7^GpppA caps are generated by the host and are used by the viral polymerase for transcription priming. Indeed, cellular decapping enzymes are known to be present in the nucleus and could provide cap structures although they are described to produce m^7^GDP or GpppN rather than m^7^GpppN [48]–[50]. However this suggestion is not consistent with the work of [25] who demonstrated reconstitution of *in vitro* cap-snatching activity of vRNA depleted THOV vRNPs, although not with highly purified, recombinant proteins.

Unfortunately our attempts to test point mutants in the two domains in minireplicon assays were inconclusive, since mutation of a conserved arginine in the putative ligand binding pocket of the central PB2 domain had little effect on polymerase activity whilst mutation of a conserved aspartate in the PA-Nter domain caused a loss of activity possibly by misfolding of the domain. Thus at this stage we are unable to elucidate the exact mechanism of cap-snatching by THOV polymerase. Further studies should focus on the reconstitution of the recombinant trimeric polymerase complex of THOV followed by more detailed mechanistic studies of its putative cap-snatching activity.

It is interesting to look at the putative cap-binding and endonuclease domains of orthomyxoviruses other than influenza or thogotoviruses in relation to their reported cap-snatching activity. For the case of the isavirus, infectious salmon anaemia virus, heterogeneous sequences of 8–18 nts length have been detected at the 5′ end of the viral mRNAs [51], suggesting an influenza like mechanism of cap-snatching. This is consistent with the sequence alignment that suggests an intact endonuclease with key metal binding and catalytic residues being conserved in isa- and IAV PA (Figure S1 in File S1). However, the presumed PB2-like subunit of the isavirus polymerase could not be aligned with that of other orthomyxoviruses [52], leaving it unclear if a cap-binding activity exists in this subunit. Interestingly, a similar situation arises with bunya and arenaviruses, which perform cap-snatching and possess an influenza-like endonuclease at the N-terminal extremity of their L proteins (polymerase) [12], [13]. However neither bunya nor arenavirus L proteins contain sequences homologous to the PB2 cap-binding domain and to date it is not clear whether and if so where these L proteins interact with the cap structure. It has also been reported that the recently identified tick borne orthomyxovirus, Quaranfil virus, has cap-snatching behaviour similar to IAV with 9–11 heterogeneous nucleotides at the 5′ end of the viral mRNAs, although other features of this virus are more like THOV [2]. This is consistent with the Quaranfil central PB2 domain exhibiting conservation of key cap-binding residues as in IAV (Figure S3 in File S1). Similarly the PA-Nter of Quaranfil conserves all the residues required for divalent cation binding as in IAV (Figure S1 in File S1). However the catalytic lysine is not found at the same position, but may occur elsewhere in the domain, as observed in other endonucleases such as those of bunyaviruses [12].

In conclusion, our data show that despite overall structural similarity, the putative cap-snatching domains of thogotoviruses have very different biochemical properties than their counterparts in other orthomyxoviruses, notably IAV, and casts serious doubt on whether they are in fact active in cap-snatching. Thogotoviruses seem to have evolved a different strategy to gain access to the cap structure required for their mRNAs, possibly reflecting their adaptation to a different host species. Further work is clearly required to resolve the enigma of how Thogotoviruses initiate transcription.

### Data Deposition

Structure factors and co-ordinates have been deposited in the wwPDB as follows:

DHOV PA-Nter 4CGS

THOV PA-Nter form 1 4CGX

THOV PA-Nter form 2 4CHC

THOV central PB2 domain form 1 4CHE

THOV central PB2 domain form 2 4CHF

THOV PB2 627 domain 4CHD

## Supporting Information

File S1 **Combined file of supporting information.**
(PDF)
