# Supplementary material for: Comparative Structural and Functional Analysis of Orthomyxovirus Polymerase Cap-Snatching Domains
Source: PLoS One. 2014 Jan 15;9(1):e84973. doi: 10.1371/journal.pone.0084973 (PMC3893164; doi:10.1371/journal.pone.0084973)
Supplement: File S1 — Combined file of supporting information. (PDF) [file pone.0084973.s001.pdf]

Comparative structural and functional analysis of orthomyxovirus polymerase cap-snatching domains

Supplementary Information

Delphine Guilligay[1,2,3], Jan Kadlec[3], Thibaut Crépin[1,2], Thomas Lunardi[3] , Denis Bouvier[1,2,*], Georg Kochs[5], Rob W. H. Ruigrok[1,2] and Stephen Cusack[3#]

[1]Univ. Grenoble Alpes, Unit of Virus Host-Cell Interactions, F-38000 Grenoble, France

[2]CNRS, Unit of Virus Host-Cell Interactions, F-38000 Grenoble, France

[3]European Molecular Biology Laboratory, Grenoble Outstation and Unit of Virus Host-Cell Interactions , 6 rue Jules Horowitz, 38042 Grenoble, France.

[4]Institute for Virology, University Medical Center Freiburg, Hermann-Herder-Str. 11, 79104 Freiburg, Germany.

[*]Present address: Laboratoire de Chimie et Biologie des Métaux, iRTSV, UMR 5249, CEA Grenoble, France.

#Corresponding author:

Stephen Cusack, European Molecular Biology Laboratory, Grenoble Outstation, 6 rue Jules Horowitz, BP181, 38042 Grenoble Cedex 9, France

Tel. (33)476207238

Email: cusack@embl.fr

Running Head: Thogoto virus polymerase cap-snatching domains

Supplementary Figures

Figure S1. Structure based alignment of PA-Nter domains from Influenzas A, B, C, Quaranfil, Salmon anemia, Jos, Thogoto and Dhori viruses. Red triangles indicate cation binding residues and a blue triangle the catalytic lysine in IAV. These residues are conserved in Influenzas A, B and C, Salmon anemia virus and Quaranfil virus (except catalytic lysine) but not in the thogotoviruses THOV, JOSV and DHOV.

Figure S2. A. Superposition of the two copies of THOV PA-Nter in the crystallographic asymmetric unit (coloured in blue and red) showing different positions of the region 72-89, including helix α3 and the following loop (coloured respectively in cyan and yellow). **B**. Packing arrangement of the two copies around the crystallographic 2-fold axis.

Figure S3. Thermal denaturation curves of THOV and DHOV PA-Nter for various concentrations of Mg^{2+} or Mn^{2+}. Note that in the absence of ions, DHOV PA-Nter is considerably more stable than THOV PA-Nter (respectively 41 and 53 °C). Unlike for IAV [1,2], no cation dependent thermal stabilization is detected and indeed cation induced destabilization is observed for THOV PA-Nter.

Figure S4. Structure based alignment of central PB2 domain domains from Influenzas A, B, C, Quaranfil, Jos, Thogoto and Dhori viruses. Blue triangles indicate residues important for cap-binding in influenza A. Cap-binding residues are conservatively substituted in Influenzas B, C and Quaranfil but not in the Thogotoviruses THOV, DHOV and JOSV. Red triangles indicate THOV residues that would clash with bound m^7GTP (Met328 and Arg344, Figure 3C). One of these, Arg344, is conserved in DHOV and JOSV.

Figure S5. m^7GTP binding to IAV (left) and THOV (right) central PB2 domains measured by isothermal titration calorimetry. m^7GTP binds to IAV cap-binding domain with a Kd of 2.7 mM but not to the THOV domain.

Figure S6. Ribbon diagrams (top) and corresponding electrostatic surfaces of the IAV (left) and THOV (right) PB2 627 domains. The IAV domain has an extensive and homogeneous positively charged (blue) surface whereas the THOV domain has some negatively charged regions (red).

Figure S7. Structure based alignment of PB2 627 domains from Influenzas A, B, C, Quaranfil, Jos, Thogoto and Dhori viruses. The green asterisk indicates the species specific residue, Lys627 in human IAV.

1. Dias A, Bouvier D, Crepin T, McCarthy AA, Hart DJ, et al. (2009) The cap-snatching endonuclease of influenza virus polymerase resides in the PA subunit. Nature 458: 914-918.

2. Crepin T, Dias A, Palencia A, Swale C, Cusack S, et al. (2010) Mutational and metal binding analysis of the endonuclease domain of the influenza virus polymerase PA subunit. J Virol 84: 9096-9104.

Figure S1

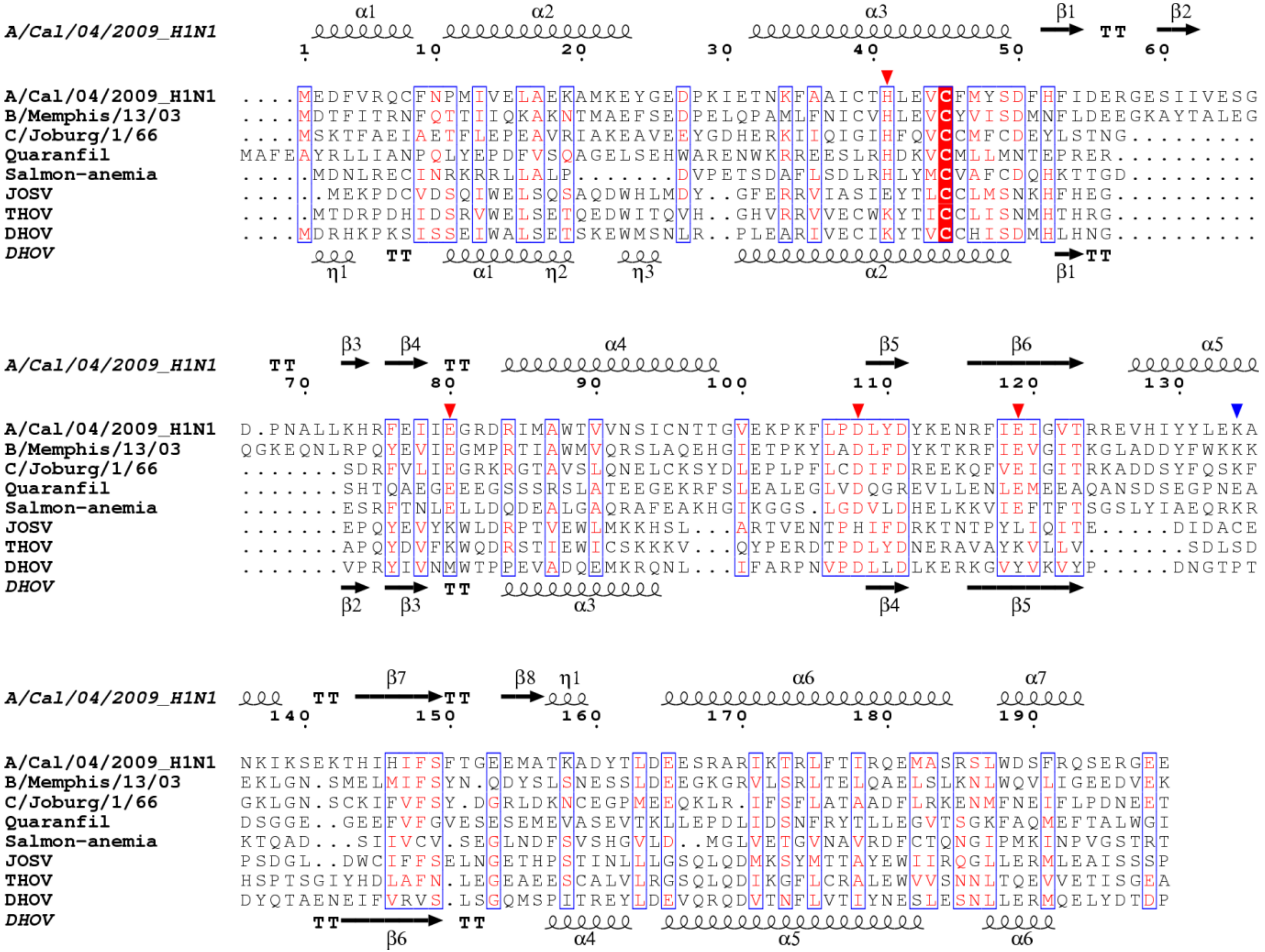
A/Cal/04/2009_H1N1
B/Memphis/13/03
C/Joburg/1/66
Quaranfil
Salmon-anemia
JOSV
THOV
DHOV
A/Cal/04/2009_H1N1 ....MEDFVRQCFNPMIVELAEKAMKEYGEDPKIETNKFAAICTHLEVCFMYSDFHFIDERGESIIVESG
B/Memphis/13/03 ....MDTFITRNFQTTIIQKAKNTMAEFSEDPELQPAMLFNICVHLEVCYVISDMNFLDEEGKAYTALEG
C/Joburg/1/66 ....MSKTFAEIAETFLEPEAVRIAKEAVEEYGDHERKIIQIGIHFQVCCMFCDEYLSTNG.........
Quaranfil MAFEAYRLLIANPQLYEPDFVSQAGELSEHWARENWKRREESLRHDKVCMLLMNTEPRER..........
Salmon-anemia .....MDNLRECINRKRRLLALP.......DVPETSDAFLSDLRHLYMCVAFCDQHKTTGD.........
JOSV ......MEKPDCVDSQIWELSQSAQDWHLMDY..GFERRVIASIEYTLCCLMSNKHFHEG.........
THOV ......MTDRPDHIDSRVWELSETQEDWITQVH..GHVRRVVECWKYTICCLISNMHTHRG.........
DHOV ....MDRHKPKSISSEIWALSETSKEWMSNLR..PLEARIVECIKYTVCCHISDMHLHNG.........
A/Cal/04/2009_H1N1 D.PNALLKHRFEIIEGRDRIMAWTVVNSICNTTGVEKPKFLPDLYDYKENRFIEIGVTRREVHIYYLEKA
B/Memphis/13/03 QGKEQNLRPQYEVIEGMPRTIAWMVQRSLAQEHGIETPKYLADLFDYKTKRFIEVGITKGLADDYFWKKK
C/Joburg/1/66 .......SDRFVLIEGRKRGTAVSLQNELCKSYDLEPLPFLCDIFDREEKQFVEIGITRKADDSYFQSKF
Quaranfil .......SHTQAEGEEEGSSSRSLATEEGEKRFSLEALEGLVDQGREVLLENLEMEEAQANSDSEGPNEA
Salmon-anemia .......ESRFTNLELLDQDEALGAQRAFEAKHGIKGGS.LGDVLDHELKKVIEFTFTSGSLYIAEQRKR
JOSV .......EPQYEVYKWLDRPTVEWLMKKHSL...ARTVENTPHIFDRKTNTPYLIQITE.....DIDACE
THOV .......APQYDVFKWQDRSTIEWICSKKKV...QYPERDTPDLYDNERAVAYKVLLV.......SDLSD
DHOV .......VPRYIVNMWTPPEVADQEMKRQNL...IFARPNVPDLLDLKERKGVYVKVYP.....DNGTPT
A/Cal/04/2009_H1N1 NKIKSEKTHIHIFSFTGEEMATKADYTLDEESRARIKTRLFTIRQEMASRSLWDSFRQSERGEE
B/Memphis/13/03 EKLGN.SMELMIFSYN.QDYSLSNESSLDEEGKGRVLSRLTELQAELSLKNLWQVLIGEEDVEK
C/Joburg/1/66 GKLGN.SCKIFVFSY.DGRLDKNCEGPMEEQKLR.IFSFLATAADFLRKENMFNEIFLPDNEET
Quaranfil DSGGE..GEEFVFGVESESEMEVASEVTKLLEPDLIDSNFRYTLLEGVTSGKFAQMEFTALWGI
Salmon-anemia KTQAD...SIIVCV.SEGLNDFSVSHGVLD..MGLVETGVNAVRDFCTQNGIPMKINPVGSTRT
JOSV PSDGL..DWCIFFSELNGETHPSTINLLLGSQLQDMKSYMTTAYEWIIRQGLLERMLEAISSSP
THOV HSPTSGIYHDLAFN.LEGEAEESCALVLRGSQLQDIKGFLCRALEWVVSNNLTQEVVETISGEA
DHOV DYQTAENEIFVRVS.LSGQMSPITREYLDEVQRQDVTNFLVTIYNESLESNLLERMQELYDTDP
DHOV

Figure S2

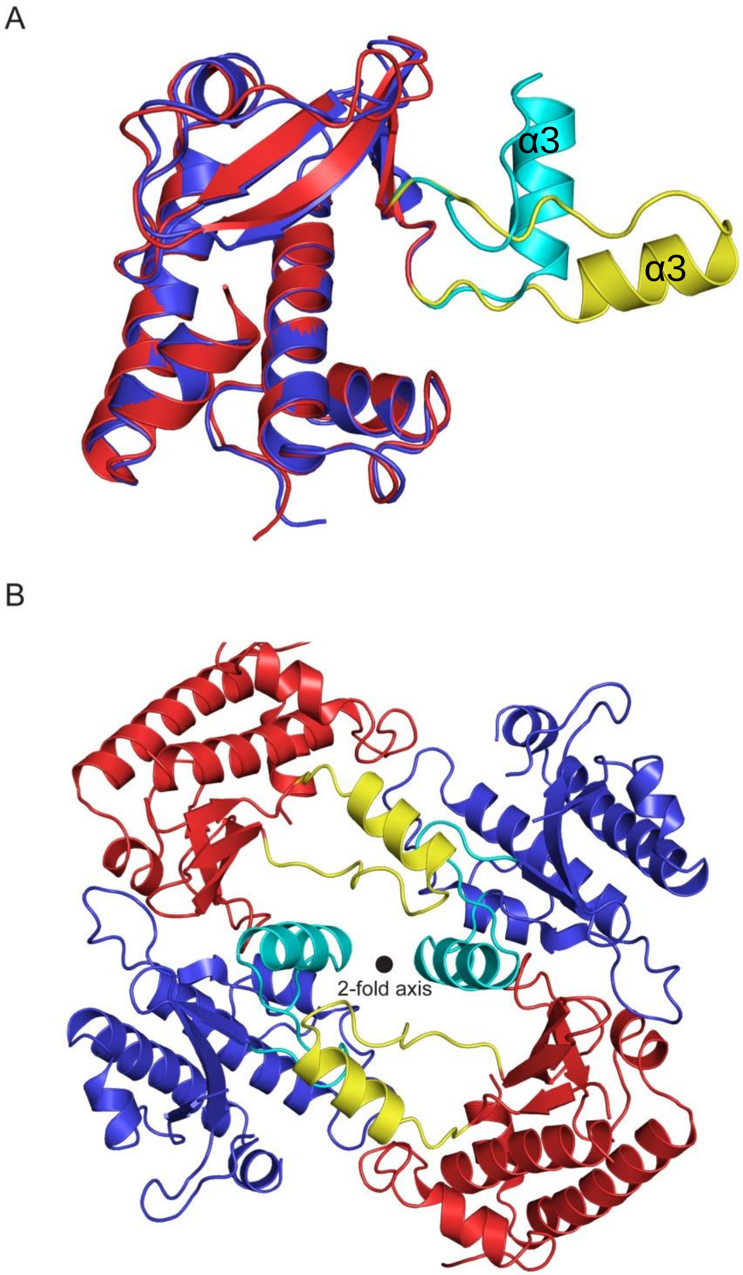

Figure S3

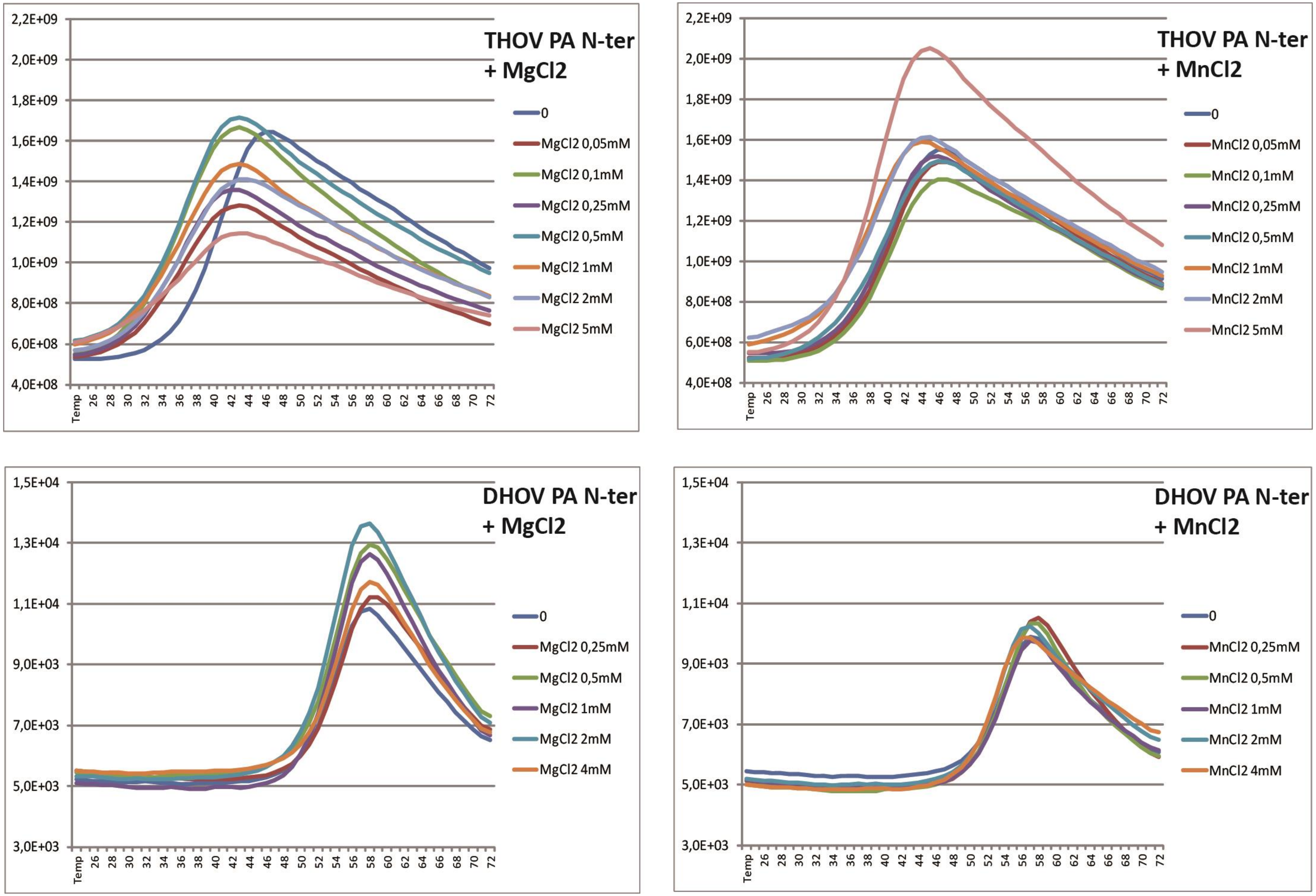
THOV PA N-ter
+ MgCl2
0
MgCl2 0,05mM
MgCl2 0,1mM
MgCl2 0,25mM
MgCl2 0,5mM
MgCl2 1mM
MgCl2 2mM
MgCl2 5mM
2,2E+09
2,0E+09
1,8E+09
1,6E+09
1,4E+09
1,2E+09
1,0E+09
8,0E+08
6,0E+08
4,0E+08
Temp 26 28 30 32 34 36 38 40 42 44 46 48 50 52 54 56 58 60 62 64 66 68 70 72
THOV PA N-ter
+ MnCl2
0
MnCl2 0,05mM
MnCl2 0,1mM
MnCl2 0,25mM
MnCl2 0,5mM
MnCl2 1mM
MnCl2 2mM
MnCl2 5mM
DHOV PA N-ter
+ MgCl2
0
MgCl2 0,25mM
MgCl2 0,5mM
MgCl2 1mM
MgCl2 2mM
MgCl2 4mM
1,5E+04
1,3E+04
1,1E+04
9,0E+03
7,0E+03
5,0E+03
3,0E+03
DHOV PA N-ter
+ MnCl2
0
MnCl2 0,25mM
MnCl2 0,5mM
MnCl2 1mM
MnCl2 2mM
MnCl2 4mM

Figure S4

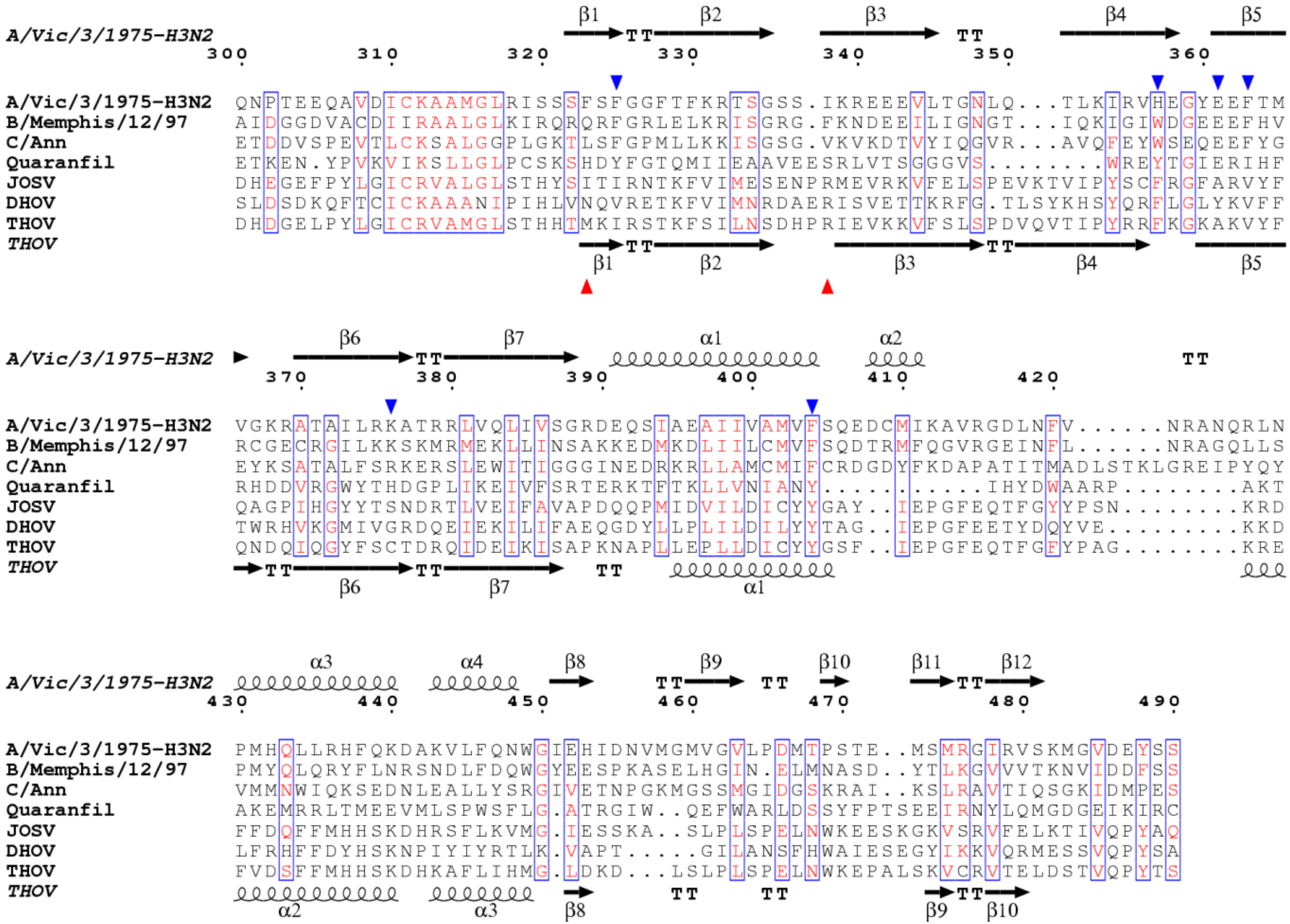
A/Vic/3/1975-H3N2
β1 β2 β3 β4 β5 TT
300 310 320 330 340 350 360
A/Vic/3/1975-H3N2 QNPTEEQAVDICKAAMGLRISSSFSFGGFTFKRTSGSS.IKREEEVLTGNLQ...TLKIRVHEGYEEFTM
B/Memphis/12/97 AIDGGDVACDIIRAALGLKIRQRQRFGRLELKRISGRG.FKNDEEILIGNGT...IQKIGIWDGEEEFHV
C/Ann ETDDVSPEVTLCKSALGGPLGKTLSFGPMLLKKISGSG.VKVKDTVYIQGVR...AVQFEYWSEQEEFYG
Quaranfil ETKEN.YPVKVIKSLLGLPCSKSHDYFGTQMIIEAAVEESRLVTSGGGVS........WREYTGIERIHF
JOSV DHEGEFPYLGICRVALGLSTHYSITIRNTKFVIMESENPRMEVRKVFELSPEVKTVIPYSCFRGFARVYF
DHOV SLDSDKQFTCICKAAANIPIHLVNQVRETKFVIMNRDAERISVETTKRFG.TLSYKHSYQRFLGLYKVFF
THOV DHDGELPYLGICRVAMGLSTHHTMKIRSTKFSILNSDHPRIEVKKVFSLSPDVQVTIPYRRFKGKAKVYF
THOV
β1 β2 β3 β4 β5 TT
A/Vic/3/1975-H3N2
β6 β7 α1 α2 TT
370 380 390 400 410 420
A/Vic/3/1975-H3N2 VGKRATAILRKATRRLVQLIVSGRDEQSIAEAIIVAMVFSQEDCMIKAVRGDLNFV......NRANQRLN
B/Memphis/12/97 RCGECRGILKKSKMRMEKLLINSAKKEDMKDLIILCMVFSQDTRMFQGVRGEINFL......NRAGQLLS
C/Ann EYKSATALFSRKERSLEWITIGGGINEDRKRLLAMCMIFCRDGDYFKDAPATITMADLSTKLGREIPYQY
Quaranfil RHDDVRGWYTHDGPLIKEIVFSRTERKTFTKLLVNIANY...........IHYDWAARP........AKT
JOSV QAGPIHGYYTSNDRTLVEIFAVAPDQQPMIDVILDICYYGAY..IEPGFEQTFGYYPSN........KRD
DHOV TWRHVKGMIVGRDQEIEKILIFAEQGDYLLPLILDILYYTAG..IEPGFEETYDQYVE.........KKD
THOV QNDQIQGYFSCTDRQIDEIKISAPKNAPLLEPLLDICYYGSF..IEPGFEQTFGFYPAG........KRE
THOV
TT β6 β7 α1
A/Vic/3/1975-H3N2
α3 α4 β8 β9 β10 β11 β12 TT
430 440 450 460 470 480 490
A/Vic/3/1975-H3N2 PMHQLLRHFQKDAKVLFQNWGIEHIDNVMGMVGVLPDMTPSTE..MSMRGIRVSKMGVDEYSS
B/Memphis/12/97 PMYQLQRYFLNRSNDLFDQWGYEESPKASELHGIN.ELMNASD..YTLKGVVVTKNVIDDFSS
C/Ann VMMNWIQKSEDNLEALLYSRGIVETNPGKMGSSMGIDGSKRAI..KSLRAVTIQSGKIDMPES
Quaranfil AKEMRRLTMEEVMLSPWSFLG.ATRGIW..QEFWARLDSSYFPTSEEIRNYLQMGDGEIKIRC
JOSV FFDQFFMHHSKDHRSFLKVMG.IESSKA..SLPLSPELNWKEESKGKVSRVFELKTIVQPYAQ
DHOV LFRHFFDYHSKNPIYIYRTLK.VAPT.....GILANSFHWAIESEGYIKKVQRMESSVQPYSA
THOV FVDSFFMHHSKDHKAFLIHMG.LDKD...LSLPLSPELNWKEPALSKVCRVTELDSTVQPYTS
THOV
α2 α3 β8 TT β9 β10

Figure S5

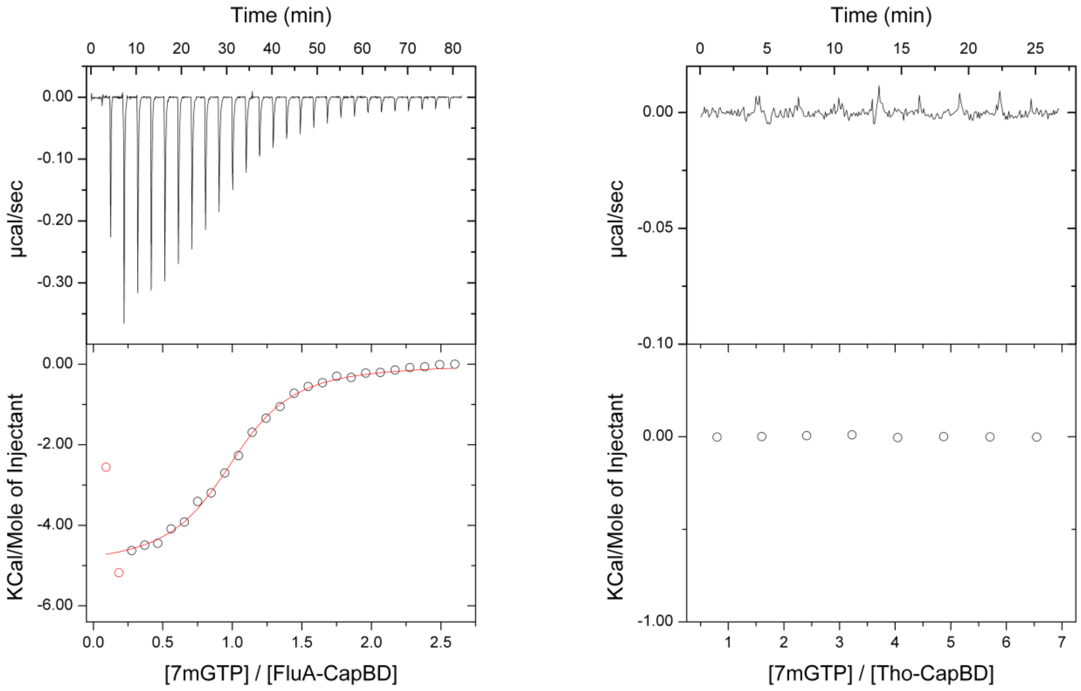

IAV

Kd = 2.7 µM

THOV

No binding

Figure S6

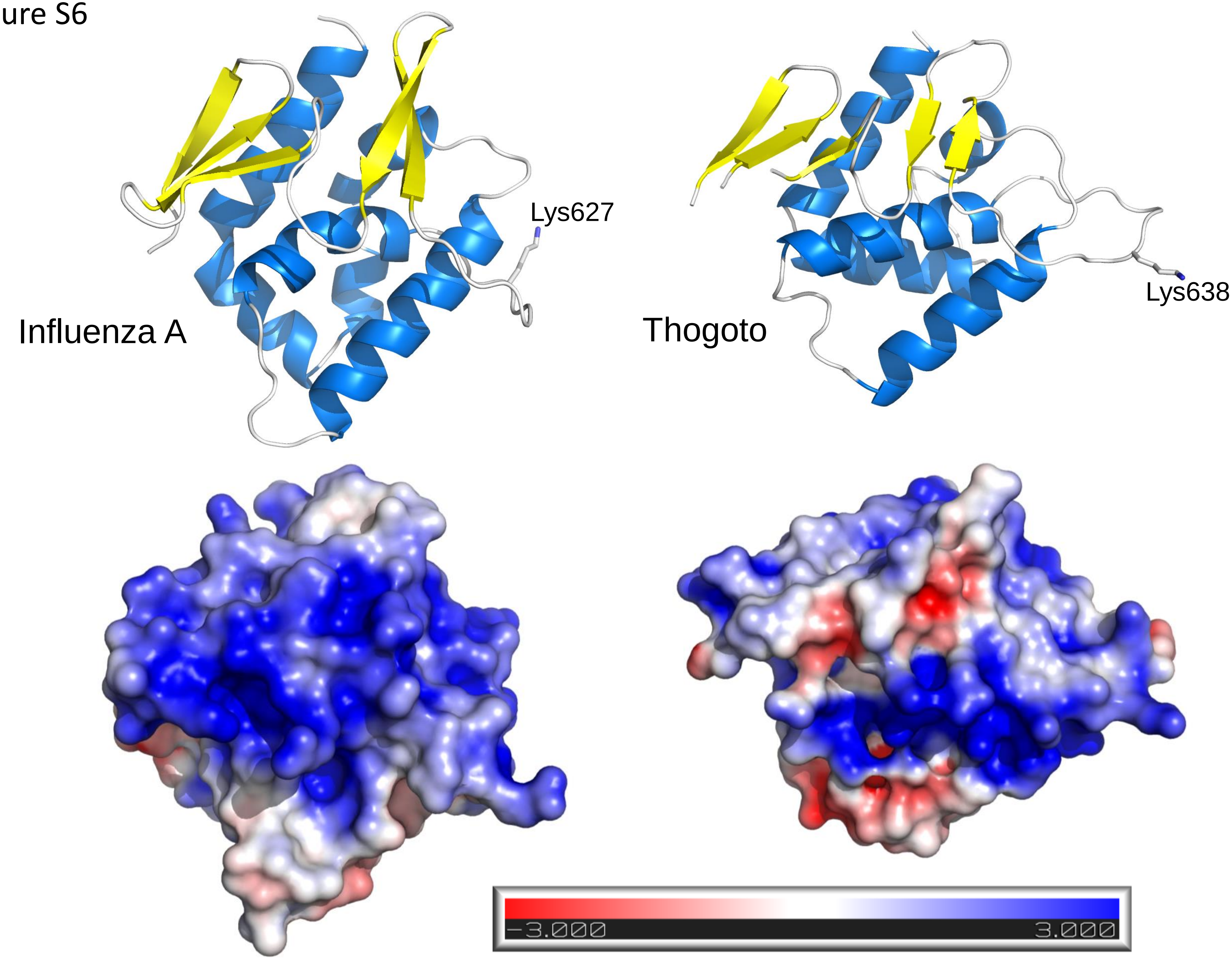

Figure S7

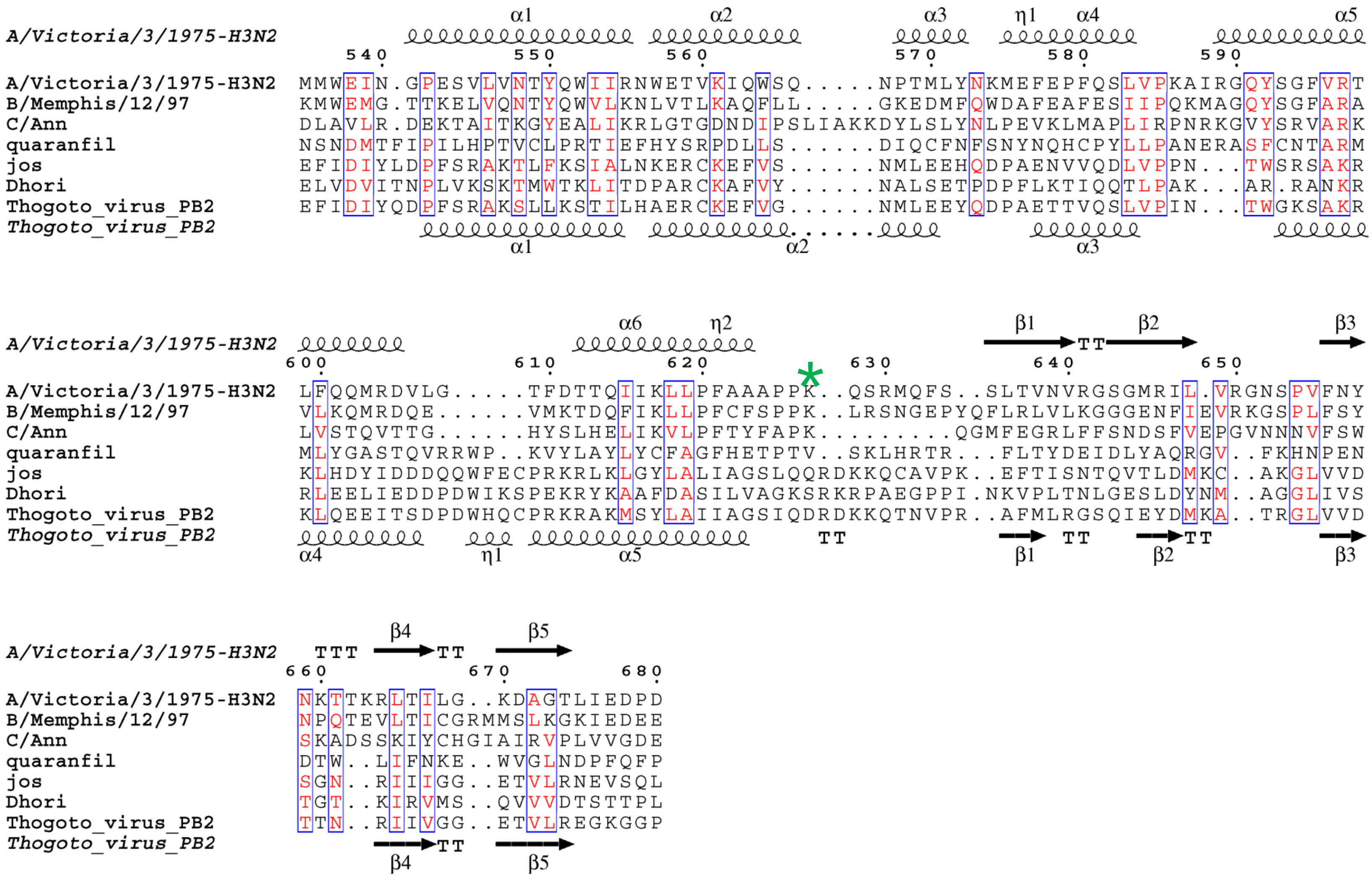
A/Victoria/3/1975-H3N2
α1 α2 α3 η1 α4 α5
540 550 560 570 580 590
A/Victoria/3/1975-H3N2 MMWEIN.GPESVLVNTYQWIIRNWETVKIQWSQ.....NPTMLYNKMEFEPFQSLVPKAIRGQYSGFVRT
B/Memphis/12/97 KMWEMG.TTKELVQNTYQWVLKNLVTLKAQFLL.....GKEDMFQWDAFEAFESIIPQKMAGQYSGFARA
C/Ann DLAVLR.DEKTAITKGYEALIKRLGTGDNDIPSLIAKKDYLSLYNLPEVKLMAPLIRPNRKGVYSRVARK
quaranfil NSNDMTFIPILHPTVCLPRTIEFHYSRPDLLS......DIQCFNFSNYNQHCPYLLPANERASFCNTARM
jos EFIDIYLDPFSRAKTLFKSIALNKERCKEFVS......NMLEEHQDPAENVVQDLVPPN...TWSRSAKR
Dhori ELVDVITNPLVKSKTMWTKLITDPARCKAFVY......NALSETPDPFLKTIQQTLPAK...AR.RANKR
Thogoto_virus_PB2 EFIDIYQDPFSRAKSLLKSTILHAERCKEFVG......NMLEEYQDPAETTVQSLVPIN...TWGKSAKR
Thogoto_virus_PB2
α1 α2 α3
A/Victoria/3/1975-H3N2
α6 η2 β1 TT β2 β3
600 610 620 630 640 650
*
A/Victoria/3/1975-H3N2 LFQQMRDVLG.....TFDTTQIIKLLPFAAAPPK..QSRMQFS..SLTVNVRGSGMRIL.VRGNSPVFNY
B/Memphis/12/97 VLKQMRDQE......VMKTDQFIKLLPFCFSPPK..LRSNGEPYQFLRLVLKGGGENFIEVRKGSPLFSY
C/Ann LVSTQVTTG......HYSLHELIKVLPFTYFAPK.........QGMFEGRLFFSNDSFVEPGVNNNVFSW
quaranfil MLYGASTQVRRWP..KVYLAYLYCFAGFHETPTV..SKLHRTR...FLTYDEIDLYAQRGV..FKHNPEN
jos KLHDYIDDDQQWFECPRKRLKLGYLALIAGSLQQRDKKQCAVPK..EFTISNTQVTLDMKC..AKGLVVD
Dhori RLEELIEDDPDWIKSPEKRYKAAFDASILVAGKSRKRPAEGPPI.NKVPLTNLGESLDYNM..AGGLIVS
Thogoto_virus_PB2 KLQEEITSDPDWHQCPRKRAKMSYLAIIAGSIQDRDKKQTNVPR..AFMLRGSQIEYDMKA..TRGLVVD
Thogoto_virus_PB2
TT TT TT
α4 η1 α5 β1 β2 β3
A/Victoria/3/1975-H3N2
TTT β4 TT β5
660 670 680
A/Victoria/3/1975-H3N2 NKTTKRLTILG..KDAGTLIEDPD
B/Memphis/12/97 NPQTEVLTICGRMMSLKGKIEDEE
C/Ann SKADSSKIYCHGIAIRVPLVVGDE
quaranfil DTW..LIFNKE..WVGLNDPFQFP
jos SGN..RIIIGG..ETVLRNEVSQL
Dhori TGT..KIRVMS..QVVVDTSTTPL
Thogoto_virus_PB2 TTN..RIIVGG..ETVLREGKGGP
Thogoto_virus_PB2
TT
β4 β5
